# Extra-Limites

**Published:** 1826-10-01

**Authors:** 


### EXTRA-LIMITES.

*Observations on Fever as it has prevailed at Smyrna during* 1825—26.
By JOHN CUTHBERT CLARKE, M.D.

The pathology of fever is involved in much obscurity, and its symptoms subject to many modifications, arising from the peculiarities of climate, and the influence of different localities. The disease is of such constant occurrence in one or other of its forms, and so diversified in its progress by the irregularities of the epidemic constitution and other causes, as to have furnished abundant materials for reflection, and ample exercise for the speculative and ingenious of every age and country. Abilities and genius of the first order have been employed in its investigation; yet, few diseases, I will venture to say, continue more effectually to baffle research, although authorities, with whom it is not safe to differ, have professed, and are believed to have unveiled, its hidden mysteries, and to have cleared the path of all obstacles to the approach of the uninitiated.

Those who have seen much of fever under its severer, more uncommon, and more concentrated character will, I apprehend, be found to be the most moderate in their assumptions as to its local habitation, its nature, or essence, and least sanguine as to the adaptation of any one unvarying or unerring line of conduct to its treatment. This, at least, is the impression which an acquaintance with the disease in different climates has produced on my mind, and, if I do not mistake, a similarly subdued tone will be found to be not uncommon amongst those whose nosological security has been disturbed by striking deviations from the ordinary process of febrile action. It is not my intention, however, to attempt to reconcile discrepancies, or to enter into the merits of the various and conflicting theories to which, even in my own time, this interesting and prolific subject has given birth. Neither do I presume to add to the confusion which already obtains, by any attempt at nosological improvement. My object is the more humble one, of endeavouring to record the history of fever as it has presented itself, and as it has fallen under my observation in this place, during the last twelve months. Its features have been sufficiently remarkable; they have been (to me at least) new, and although not unknown to the practitioners of the place, several of them, men who have passed great part of a long life in the Levant, they have been little investigated, and have been regarded as peculiarly fatal and irremediable. From the authorities also within my reach, I have been able to obtain but little information, excepting such as is to be derived from the vague descriptions of the French writers, and their allusions to the older authors, the somewhat analogous congestive typhus of Dr. Armstrong, and various observations contained in the works of the illustrious Jackson, to whose unwearied zeal, and philanthropic labours in this department of human suffering, science owes so much, and the gratitude of his fellow men still more. Under these circumstances, I am led to hope that an attempt to describe an uncommon form of disease, (which, as far as I know, has no place in any English classification, and no parallel that I am acquainted with, in its distinguishing symptoms—the diminution of temperature, and torpor of the circulating system, excepting in the account communicated by Dr. Dickson to Dr. J. Johnson, and inserted in his valuable work on Tropical Diseases, relative to a fatal fever, which afflicted the garrison of Mariegalanté in 1803,) will not be found to be devoid of interest, especially to those whose destinies lead them into countries where fever assumes its most appalling aspects.

It may be excusable to premise, that the city of Smyrna is situated at the head of a deep gulf, bounded nearly on all sides by a bold and deeply indented outline of mountain scenery, in some parts of considerable elevation. The approach to the city by sea is somewhat circuitous and winding, in consequence of a long narrow tongue of land, which is gradually increasing its dimensions by the detritus of the Hermus, from the mouth of which, it extends across the gulf, leaving a narrow channel between its extremity and a projecting point of low land which approaches it from the opposite side. The city is built on the broad base of a steep hill, crowned by the ancient citadel, and covers a much greater extent of ground than would be imagined by any one approaching it from the sea, and unacquainted with the undulations, and irregularities of the surface which intervenes between it and the steep acclivities, on which stands the Turkish quarter. This quarter is inhabited by a dense population of Mahometans and Jews, who have wisely entrenched themselves in the most healthy district, leaving the lower space to the Christian population, including under that general denomination, Armenians, Greeks, Catholics, and a few Protestants. Parallel with the margin of the sea is the Frank Street, which is built entirely on alluvial formation, and may be said to stand over a bed of water, which is met with abundantly at the depth of a few feet. To the northward and eastward of the town, a long sandy point forms one side of the entrance of a deep inlet or bight, the southern shore of which, is partly under cultivation and partly marsh. A deep valley through which flows the Meles, now an insignificant stream, and which has its outlet in this marsh, winds round the hill on which the city is placed, and in these situations fever, at particular seasons, is not unfrequent.

The climate is subject to considerable vicissitude; the summer heat is oppressive, and would be intolerable but for the regular succession of land and sea breezes, the thermometer, in the months of July and August not unfrequently ranging as high as 82° and 88° in the shade; and occasionally in southerly winds, reaches 95° and 96°, while the cold of winter is peculiarly piercing and keen, although the thermometer does not indicate the degree of cold which is perceptible by the living body. Notwithstanding all these sources of disease, however, the climate can scarcely be said, in ordinary seasons, to be an unhealthy one. Autumnal fevers of a distinctly remittent type, severe in degree it is true, but by no means intractable under proper management, were frequent during the first years of my residence here, yet they were generally traceable to the influence of miasmata, and particularly to those of the plain of the Cayster in the neighbourhood of Ephesus, which, like other ancient and deserted cities, conceals amongst its ruins, a severe penalty on those who visit it at improper seasons. Of late years, however, comparatively few travellers have ventured thither, and that source of disease being withdrawn, fevers of indigenous growth and unusual character have taken its place. The political evils to which the country has been exposed, and in which all classes have more or less participated, have no doubt had their share in the production of disease, which has never, however, assumed a contagious character, and is clearly of domestic origin.

I had occasionally, in consultation with others, but more particularly since the commencement of the Greek insurrection, had opportunities of observing the first form of fever in question, namely, the "fièvre pernicieuse algide" of the French nosologists, amongst Greeks, Armenians, and other subjects of the Porte, a class of people, on whom the depressing passions have amazing influence; who, either from fears for their own, or their friends' safety, or the equally alarming fears for property, have been attacked by this formidable disease. In such cases, the circulation suddenly forsook the surface, an icy coldness pervaded the whole frame, the pulse was in many instances, scarcely perceptible, more frequently it was altogether abolished. The sensorial functions gradually partook of the benumbing influence, and the powers of life were surrendered without a single rallying effort. I seldom had an opportunity of seeing these cases a second time, but I have reason to believe that they were almost uniformly fatal. Amongst those which subsequently occurred in my own practice, and which I had it in my power to watch from the beginning, the greater part occurred last summer, and the symptoms which were alike, appeared in nearly the following order. After two or three days of torpor and indisposition, the patient is attacked by chilliness, succeeded by increase of heat, head-ach, muscular and dorsal pains, lassitude and prostration of strength. The pulse is accelerated, but not remarkably increased or diminished in volume. He complains of thirst, and general uneasiness, together with such other symptoms as are common to the invasion of all fevers. There is nothing, however, either in the appearance of the patient, or in the nature of the symptoms which can indicate the approach of the remarkable derangement of balance which is about to take place. I have observed two persons, of the same age, of similar habits, living in the same apartment, and who were attacked about the same time, in whom the leading symptoms were alike, and who were both subject to nearly the same treatment but in only one of whom the algid symptoms ensued; while, in the other, the course of the disease was that of the more open and regular fevers of the country. About the second or third day, but sometimes earlier, the extremities are found to be cold, and, on examining the trunk, and other parts of the body, the same extraordinary diminution of temperature is found to have taken place. A change, however, of which the patient is quite unconscious until he is informed of it by those about him. There is no shivering, nor is the slightest sensation of cold complained of; the pulse, if felt at all, is thready and exceedingly rapid, and disappears under the slightest pressure of the finger. There is more usually, a total want of pulsation in all the tangible vessels, and even in the region of the heart. The countenance, in some cases, is livid, and betrays unspeakable anguish; the features are withered and shrunk. The intellectual powers are, for the most part, but little impaired; and, there is frequently a command of muscular exertion, a calm composure and self-possession which would lead any one, unacquainted with the patient's situation, to believe him free from complaint; but, when the fingers are applied to the wrist, and the pulse is sought in vain, when the more than deadly coldness of the surface is perceived, the illusion vanishes. In this state of equivocal existence, he continues from 10 to 18 or 24 hours, and, in some cases, much longer, when, if he survives the first paroxysm, by the persevering application of remedies, and the re-active energies of the system, an increase of temperature takes place, which gradually acquires different degrees of intensity, according to the capacity of the powers of life and the nature of the means employed, and occasionally requires to be moderated. After an uncertain interval, however, the same appearances are renewed, and either carry the patient off, or are repeated with diminished violence until the healthy action is re-established.

The following case may serve as a specimen of the disease, which it has been my aim to describe. Others, in which the symptoms were, in some respects, still more remarkable, might have been added; but I have been deterred from doing so by unwillingness to occupy, unnecessarily, the time of others, or to exceed the limits which I had assigned to this paper. With regard to the remedial means employed, I have nothing new to propose, and am willing to ascribe the successful treatment of the disease, chiefly, to the unremitted application of such resources as experience has shewn to be best adapted to fevers of a congestive character or of irregular excitement.

*Case.* A gentleman who had been, for a considerable time, employed in antiquarian research amongst the islands of the Archipelago, and on the coast of Asia Minor, arrived here on the evening of the 25th August, 1825. He had been indisposed, and confined to bed for several days, and had taken a laxative medicine the preceding morning. I found him labouring under considerable depression of spirits, and restlessness, his pulse was quick and feeble, his skin of nearly natural heat;—he was thirsty and anxious, yet he made no particular complaint, and there was no prominent, or urgent symptom in the case. His mind was, therefore, tranquillized as much as possible, and I prescribed for him a camphorated, saline mixture, with ten grains of calomel at bed time. 26th. His pulse had gained considerably in strength his skin was moderately warm, his bowels had been acted on by the calomel, and he felt, altogether, more comfortable than on the preceding day; his tongue was moist, thirst was moderate, and he had had some hours of refreshing sleep. A powder, composed of pulv. Jacobi, g. ij. calomel, g. iij. pulv. tragacanth. co. g. iv. was administered every four hours, and he was left at night better in every respect, and in no unpromising condition. Shortly afterwards, however, the algid symptoms appeared, and on being called, I found him pulseless and cold. There were still some faint remains of warmth in the axillæ, but these quickly disappeared, and the whole body soon communicated to the hand, a peculiarly repulsive sensation of cold. No pulse could be perceived in the crural, temporal, or carotid arteries, nor in the region of the heart was any action perceptible; yet, to my questions he replied, that he felt comfortable, and complained only of thirst; his stomach continued retentive, and his bowels were readily relieved by a stimulating enema. A blister was now applied to each leg, a very large one to the epigastric region; stimulating frictions and bottles filled with hot water were applied to different parts of the body; sinapisms were placed on the soles of the feet and on the thighs. He took six grains of the sub. carb. ammoniæ, combined with opium, every three hours, together with a strong decoction of bark and valerian, in doses of two ounces every hour. Notwithstanding an assiduous application of these means, however, he was found, on the morning of the 27th, with scarcely any increase of heat; and several hours elapsed before the pulse was distinguishable at the wrist. About two P. M. the pulse and heat were both restored, the former being much accelerated. The tongue now became dry and parched, its edges and apex being exceedingly red and fiery. He complained of sensibility on pressure over the epigastrium; thirst became more urgent; the bowels were relaxed and irritable; and the pulse rose to 110. Under these circumstances 18 ounces of blood were abstracted from the arm, by which he was decidedly relieved in every respect. The decoction of bark had been exchanged in the morning for an oily demulcent mixture, and mucilaginous fluids, calculated to soothe the now excited mucous membrane of the bowels. The sub. carb. ammoniæ had been omitted, and, in consequence of head-ach, a blister was applied to the nape of the neck. About seven in the evening, a sudden reduction of temperature took place, which continued the whole of the night, but unaccompanied by shivering or the slightest sensation of cold, and did not diminish until eleven o'clock, A. M. of the following day. The same means were employed, as during the former paroxysm; the succeeding excitement was much more moderate; and he passed the day more composedly than the preceding one The cold stage returned about 6 P. M. but subsided early in the morning of the 29th. He had little increase of heat during the day, and the algid symptoms returned at a later hour; they were, however, less intense, and of shorter continuance. He was now able to continue the decoction of bark every two hours; no preternatural increase of heat followed; and the frigid symptoms returned no more. He was removed to apartments on shore on the 2d September, and continuing the decoction of bark a few days longer, his recovery was uninterrupted and complete.

It is no part of my purpose, to indulge in speculation as to the morbid cause by which the sources of vital heat, and that due co-operation between the nervous and sanguiferous systems which is believed to be essential to the maintenance of the calorific function, become thus obstructed and unhinged. The subject is connected with other questions which are involved in so much ingenious ambiguity, that I forbear to do more than to place the facts as they have occurred to me before more competent judges. "Where so many and such various chemical processes are going on as in the living body, are we justified in selecting any one of them for the purpose of explaining the production of heat?" is the question of a distinguished physiologist, and where he has acknowledged the difficulties with which this mysterious subject is beset, I may well be permitted to pause. How far the direct operation which some modern physiologists are disposed to assign the nervous system in the evolution of heat, is reconcileable with the phenomena of this remarkable disease, may perhaps be questionable, inasmuch as the integrity of the sensorial functions, and other ordinary manifestations of the nervous agency are unimpaired, while the stimulus of the arterial blood which is supposed to be necessary for the due excitement of the brain and nerves, appears to be withheld, or very much diminished, by the suspension of all cognizable arterial action. No spasmodic, gastric, or intestinal disturbance, as in the cholera of India, in which reduction of temperature is a conspicuous symptom, countenances the supposition, however probable, that the par vagum, and great sympathetic, with their numerous and important relations are mainly implicated in the chain of causes. The accumulation of blood in the venous system, in the spleen and other visceral receptacles, with its difficult transmission through the lungs, and the defective chemical change which it there undergoes, are in this disease, at least, the most prominent causes of diminished temperature. But if there is obscurity in the process by which the organs concerned in the elaboration of heat perform their functions, what new arrangement or combination is employed in the production or extrication of the mortal cold which gradually overspreads the entire surface? The subject has been treated, with what success it does not become me to decide, by M. Bailly (de Blois) in a communication inserted in the 2d vol. (new series) of the Revue Medicale, relative to a fever of the same description, which he had opportunities of observing in l'Hôpital du St. Esprit at Rome, in 1822. His pathological and physiological observations are, like the reasonings of his ingenious countrymen on such subjects, marked by considerable plausibility, yet, his deductions, or the deductions of those with whom he was associated, as is not unusual with that unequally gifted people, appear to me to have led to inefficient, if not erroneous practice; since stimuli, and the continued application of external warmth were withheld or insufficiently employed; and the result of the cases detailed, five in number, only one of whom recovered, is far from encouraging. But whatever may be the rationale of the morbid phenomena, I have acted on the broad principle of sustaining the living powers; of moderating them when roused to excess; and it is in my power to shew, that the steady application of such agents as are calculated to fulfil these indications has been successful in twelve of thirteen cases of the disease which came under my personal observation and care. This circumstance will account for the want of such post mortem examinations as might have served to illustrate the subject; and as dissection is never permitted by the people of the country, I have not been able to avail myself of the observations of others. From the same cause, I am unable to corroborate by my own observation a statement of M. Bailly relative to the evolution of heat after death; although I am assured by others, on whose accuracy I have some reliance, that such was the fact in some instances during the prevalence of the disease here; and that, in some cases, it was so freely developed as to prevent interment from taking place for some hours longer than is usual with the Greeks and other people of the country, whose custom it is to bury their dead without delay.

One case of the algid disease has already occurred to me this season, and re-action never took place. The patient sunk after lying upwards of 36 hours in the state described. Its appearance has been unusually early, and I am inclined to believe, that it and the form of fever which I will now beg leave as briefly as possible to describe, have been one and the same disease, modified by circumstances, and by the change of season. About the close of September the first-mentioned disease subsided. Fever, however, continued to occur sporadically, and began to assume a new complexion. A keen northerly wind commenced in the month of January, and continued to blow with an inveteracy unknown to the oldest inhabitant during three months. Rain fell, but very sparingly, and not in sufficient quantity to unload the drains or sewers which convey all the impurities by a rapid descent from the upper town to the sea, and which now stagnated in the level space beneath. The outlets of these drains are confined to the quarter occupied by the lower half of Frank Street, which is about three-quarters of a mile in length, and to this district the disease confined its operations, any isolated cases which occurred in other parts of the city being amongst brokers or other persons employed during the day in the business of the European merchants in the Frank Street, where it was contracted and conveyed by them to their homes, in which, however, it never became contagious. This disease commenced frequently with catarrhal symptoms; but, in all cases, its approaches were gradual and insidious; the patient being able to attend to business for several days before decided illness compelled him to retire. He was then suddenly overpowered by great languor and listlessness; the pulse was found to be quick and feeble; an increase of heat and vascular action soon followed in most cases. The pulse became full, bounding, and gradually acquired a tumultuous irregularity, which, about the third day in general, defied all calculation. Thus, it was found in the morning either entirely wanting at the wrist, or barely perceptible; some hours afterwards two or three irregular throbs were followed by a long intermission; and in the evening it was occasionally found to be unchained, although still irregular, and intermitting at longer intervals. The patient sighed deeply, and complained chiefly of a load and oppression at the præcordia; the countenance became mottled and grim; livid streaks and patches appeared on different parts of the body, with a very faint substratum of yellow, and at this stage death occasionally took place about the 4th or 5th day. In the majority of cases, however, about the 6th or 7th day, the patient's entire body was found to have assumed a deep yellow colour; the tongue became dry, contracted, and glazed. The pulse continued labouring, obstructed, and irregular for several days; the stomach, in a very few cases, became irritable, and rejected whatever was received, either as medicine or drink. Retention and suppression of urine were not unfrequent symptoms, the latter being observed chiefly amongst those cases which ended fatally. Under these circumstances, the disease extended very frequently to the 14th and 21st days, before any decided amendment could be perceived; the first indication of which, however, was afforded by the pulse becoming more regular. But the convalescence in all, even the most favourable cases, has been lingering and protracted, beyond any that I have ever met with; and the cessation of the morbid action has been so gradual and imperceptible, and attended by so little decided improvement, as, in many cases, to have kept alive the solicitude of the medical attendant and friends to unusually extended periods of febrile termination. Ophthalmia has been not an unfrequent sequel of the disease, and was obstinate in character. The yellow suffusion was obviously the result of great hepatic engorgement, and was hailed, after some acquaintance with it, as rather a favourable omen, when occurring about the 6th or 7th day, as it was generally attended by some relief of the peculiarly constrictive and oppressive sensation at the præcordia.

The treatment which I was led to adopt in this form of the disease was suggested by the strong evidences of visceral obstruction, and of a congestive state of the circulation in the great venous trunks. In the early part of the disease, the pulse was such as indicated the necessity of free sanguineous depletion; the effects, however, of this admirable remedy were, I am sorry to say, far from satisfactory. It was badly borne in every case, and a repetition of bleeding was supported still worse, the loss of a few ounces of blood being sufficient to bring on alarming lowness and syncope. Local bleeding by leeches nevertheless was advantageously substituted. Emetics in the forming stage of the disease were frequently very useful. Purgatives were always had recourse to, and, when preceded by one or two scruple doses of calomel, were productive of their usual good effects. On the constitutional effects of that medicine, however, my chief reliance was placed, particularly on the latter disease. Together, therefore, with the internal administration of calomel, I was led to join camphorated mercurial friction over the right hypochondriac region, and, without referring to the disputed point of its modus operandi, I have only to observe that its effects on the salivary apparatus have been, in this disease, such as I have been accustomed to observe them in other fevers, but particularly those of irregular excitement, or of protracted character, ordinarily connected with a solution of the disease. Whether these are to be regarded as causes or effects of that solution I do not now enquire; some experience in fever of that description, and particularly in this climate to which free depletion has been inapplicable, and in which the more energetic stimulants have been inadmissible, having so long led me to associate its appearance with that of returning health, that I fully agree with those who consider this invaluable remedy amongst the best of our therapeutic resources in fever. Blisters were applied according to circumstances, but for the most part with unsatisfactory effect, the superficial circulation being so languid, as in most instances under my observation to prevent any salutary effect from them as a remedy. They produced scarcely any vesication, and occasioned little more than the effect compared by Dr. Jackson to that of parts seared by a heated iron. The warm bath was decidedly serviceable in some cases of this and the preceding disease; but the attendant fatigue, and alarming symptoms of collapse which more than once occurred in approaching the erect posture deterred me from its further use. But whatever other means were employed, the speedy supervention of great debility and prostration in every case, soon pointed out the necessity of supporting the patient's failing strength. Bark which, in the ordinary fevers of this country, is a remedy above all praise, after the vital organs have been secured by bleeding, was here found to be inadmissible, and was abandoned, but a free use of brisk porter or cider was found to fulfil this indication to the most satisfactory extent. Some experience in the administration of the first of these articles, two years and a half ago, amongst a number of sick belonging to

H. M. S. Cambrian, placed under my care in this hospital, and afflicted with one of the worst typhoid fevers I have ever witnessed, in consequence of having conveyed a part of the wretched Turkish garrison of Napoli di Romania to this place, and who were more benefited by a liberal allowance of it than by any remedy whatever, encouraged me to extend its use to the fever in question, and I have had much reason to be satisfied with the result, which has, I am happy to say, been successful in every case of which I have had the charge.

In the early part of June, cases of this disease had become rare, and it was hoped would entirely disappear under an increase of temperature. The event, however, has been otherwise; its distinctive characters continue the same, but the march of the disease has been more rapid. It has never, in any instance, assumed the character of contagion. It has been, and continues to be, confined to a limited district, beyond which it has not strayed, unless when conveyed by persons labouring under the disease, and it has never been communicated by them to others. The mortality, considering the number attacked, has hitherto been inconsiderable; and has been chiefly amongst the Greeks, whose prejudice and pusillanimity, in sickness as well as in the ordinary affairs of life, are invincible.

JOHN CUTHBERT CLARKE.

*Smyrna, July 1st*, 1826.

